# Deep Learning on Histopathology Images for Breast Cancer Classification: A Bibliometric Analysis

**DOI:** 10.3390/healthcare10010010

**Published:** 2021-12-22

**Authors:** Siti Shaliza Mohd Khairi, Mohd Aftar Abu Bakar, Mohd Almie Alias, Sakhinah Abu Bakar, Choong-Yeun Liong, Nurwahyuna Rosli, Mohsen Farid

**Affiliations:** 1 Faculty of Computer and Mathematical Sciences, Universiti Teknologi MARA, Shah Alam 40450, Malaysia; sitishaliza3425@uitm.edu.my; 2 Department of Mathematical Sciences, Faculty of Science & Technology, Universiti Kebangsaan Malaysia, Bangi 43600, Malaysia; mohdalmie@ukm.edu.my (M.A.A.); sakhinah@ukm.edu.my (S.A.B.); lg@ukm.edu.my (C.-Y.L.); 3 Department of Pathology, Faculty of Medicine, Hospital Canselor Tuanku Muhriz, Universiti Kebangsaan Malaysia, Jalan Yaacob Latif, Bandar Tun Razak, Cheras, Kuala Lumpur 56000, Malaysia; nurwahyuna@ukm.edu.my; 4 Department of Computing and Mathematics, University of Derby, Kedleston Road, Derby DE22 1GB, UK; m.farid@derby.ac.uk

**Keywords:** breast cancer, bibliometric analysis, healthcare, medical imaging, VOSviewer

## Abstract

Medical imaging is gaining significant attention in healthcare, including breast cancer. Breast cancer is the most common cancer-related death among women worldwide. Currently, histopathology image analysis is the clinical gold standard in cancer diagnosis. However, the manual process of microscopic examination involves laborious work and can be misleading due to human error. Therefore, this study explored the research status and development trends of deep learning on breast cancer image classification using bibliometric analysis. Relevant works of literature were obtained from the Scopus database between 2014 and 2021. The VOSviewer and Bibliometrix tools were used for analysis through various visualization forms. This study is concerned with the annual publication trends, co-authorship networks among countries, authors, and scientific journals. The co-occurrence network of the authors’ keywords was analyzed for potential future directions of the field. Authors started to contribute to publications in 2016, and the research domain has maintained its growth rate since. The United States and China have strong research collaboration strengths. Only a few studies use bibliometric analysis in this research area. This study provides a recent review on this fast-growing field to highlight status and trends using scientific visualization. It is hoped that the findings will assist researchers in identifying and exploring the potential emerging areas in the related field.

## 1. Introduction

Cancer may arise from almost any part of the human body where cells start to grow uncontrollably [1]. Deaths caused by cancers keep increasing every year and are considered as the main illness globally [2,3,4]. Breast cancer is one of the top illnesses contributing to the highest death rates among women, especially in developing countries such as Melanesia, Western Africa, Australia, Micronesia/Polynesia, and the Caribbean [5]. However, it is noticeable that the percentage of breast cancer cases in Australia, Western Europe, Northern America, and Northern Europe are the highest [5,6]. Women are commonly diagnosed with breast cancer, but men, however, are not excluded [7]. The breast structure of women is mainly made up of milk ducts, lobules, and adipose tissue [8]. Breast cancer may initiate in the ducts which carry milk to the nipple or in the lobules glands, the part of the breast that produces breast milk [8,9]. Globally, the majority of breast cancers are of ductal and lobular subtypes, given that 40–75% are comprised of ductal subtypes of all reported cases [10].

Early diagnosis and treatment may benefit in preventing breast cancer from developing to the advanced cancer level. There are several medical imaging procedures for breast cancers such as mammograms (X-rays), ultrasound (sound waves/sonography), magnetic resonance imaging (MRI), and biopsy [11,12,13,14]. However, the use of breast cancer images to confirm the cancer region is only available through biopsy procedures [15]. Tissue biopsy examination is currently the clinical gold standard in cancer diagnosis. Tissue biopsy produces histopathology images that can enhance the results of breast cancer classification [16]. The basic procedure in biopsy is collecting a tissue sample from the body for further analysis by the histopathologist [17]. The tissue will be immersed in the formalin solution and planted in paraffin wax before being cut carefully, resulting in histopathology slides which then converted to images [18,19]. However, the manual procedure of biopsy analysis is tedious, time-consuming, and restricted by the quality of the histopathology image and the histopathologists’ skill [20,21]. The histopathology images are stored and analyzed using the Computer-Aided Diagnosis (CAD) system [22]. The CAD system is used to overcome the issue of classification accurateness from manual approaches [23], and machine learning techniques are required [24].

The involvement of machine learning algorithms could help to reduce the number of unnecessary biopsy images. For an image analysis, there are four important stages to be considered: (i) input, (ii) feature extraction and selection, (iii) classifier model, and (iv) classifier output. According to Nahid and Kong [8], feature extraction and representation approaches are important to produce accurate and reliable results. There are two types of features which are hand-crafted features and learned features. Expert-level knowledge is required for hand-crafted feature extraction during image analysis [25]. A predefined hand-crafted feature is important in traditional machine learning methods, such as support vector machine (SVM), Naïve Bayes, random forest (RF), and *k*-means clustering. For example, [26] used regional and localized features with SVM as a classifier to evaluate the quality of 3D images. On the other hand, wavelet transform was applied to tree-structured algorithm for automatic image grading in two datasets with different magnification factors [27]. The authors used *k*-means clustering and texture features to locate the affected regions in the segmentation process. Similarly, [28] also used the wavelet transform to extract the features from breast cancer images and SVM classifier meant for feature selection. The result indicates that the combination of SVM classifier and chain-like agent genetic algorithm (CAGA) to obtain the optimal feature set was remarkable, with an accuracy of 96.19%.

The majority of the studies are limited to a macroscopic overview of breast cancer image classification. Specific visual bibliometric analysis is relatively low. Based on the bibliometric analysis, this research aims to present updated and microscopic overview characteristics of breast cancer image classification publications. The clear and informative maps offered in this work highlight research accomplishments in the deep learning on breast cancer image classification domain, which may aid researchers and practitioners in identifying the underlying implications of authors, journals, countries, references, and research themes. The co-authorship network analysis is believed to give some insight on the intellectual collaboration and interaction between researchers. In detail, the focuses of the paper are: (i) to examine the number of papers on the rise of publications and citations on deep learning approaches published from the years 2014 to 2021, (ii) to map the co-authorship networks among countries, authors, and scientific journals, and (iii) to analyze the co-occurrence network of the authors’ keywords globally.

This paper hopes that the findings will help to initiate ideas for future research in the related field and, in turn, will benefit the patients and healthcare providers. This study is also important as guidance for researchers that are unfamiliar with deep learning but interested in its potential in breast cancer image classification, where most active researchers and recent significant research topics among authors are discovered. This study specifically highlighted the application of deep learning instead of machine learning since recently, the field has been more strongly associated with image classification. Based on the overview of the progress, it is estimated that deep learning will continue to evolve and flourish as a significant tool for image classification.

## 2. Breast Cancer Image Classification

### 2.1. Bibliometric Analysis of Breast Cancer Studies

Bibliometrics can visualize the structure of the scientific disciplines based on the bibliographic information gained from the databases [29]. Bibliometrics have been used in vast scientific areas to analyze prior studies’ trends and patterns, such as web accessibility, text mining, sustainable business, and healthcare [29,30,31,32]. Some bibliometric studies have discussed breast-cancer-related topics. Cinar [33] provided a bibliometric analysis on 2734 articles related to breast cancer focused on the nursing field from the year 2009–2018. Based on the keyword analysis, the term “breast cancer survivor” was highly cited in year 2014 to 2018, and research showed a progressive trend of breast cancer related to the nursing field within those five years. Salod and Singh [34] studied the publication trends, country collaboration, author productivity, institutional collaboration, and productive journal based on the literatures related to breast cancer in the field of machine learning.

In a recent review, Joshi et al. [35] studied machine learning methods towards breast cancer histopathology images. Machine learning is a subset of artificial intelligence which includes statistical methods that can improve and learn the information directly from data. They pointed out that there was a growing interest in machine learning and histopathology images of breast cancer. Based on keyword analysis, their study revealed that disease in female, breast cancer, deep learning, histopathology and medical imaging are the top important keywords [35]. This showed that machine learning applications offered a potential research trend towards medical images analysis. However, the final performance of image analysis relies on the pre-processing data, including hand-crafted features extraction which is hard to solve by using traditional machine learning methods [25,36]. With the technological evolution of deep learning and rapid growth research of the application in healthcare, especially breast cancer, understanding the development of deep learning has become essential.

### 2.2. Breast Cancer Image Database

Breast cancer is a common cancer type among people, especially women, around the world. An early detection of breast cancer would lead to an appropriate treatment which might increase the survival rate of affected people [35]. Hence, a well-defined database is important to measure the performance of breast cancer classification models. There are several databases that are publicly available for breast cancer diagnosis such as Mammography Image Analysis Society (MIAS), Wisconsin Breast Cancer Dataset (WBCD), Digital Database for Screening Mammography (DDSM), Breast Cancer Histopathology (BreakHis), and Breast Cancer Histology (BACH). Since deep learning is gaining the fame for its ability to process image data in hierarchical representation using nonlinear transformations [25,37], hence the histopathology images are broadly used by researchers. The BreakHis and BACH datasets were made up of histopathology images. According to Li et al. [38], BreakHis dataset is extensively used in CNN algorithms related to image classification. They propose a new CNN architecture that uses local information in the breast cancer images and extra features extraction through different dense blocks and SENet module.

The BreakHis dataset was first introduced in 2016, which comprised 7909 histopathology images collected from the P & D Laboratory, Brazil [39]. Nahid and Kong stated that after the introduction of BreakHis dataset, there were about 20 articles published within a year from 2016 to 2017. Out of the total images, 2480 were benign images and 5429 malignant images with four different magnification factors. Table 1 showed detailed image distribution based on the magnification factors 40×, 100×, 200×, and 400×. Similarly, the BACH dataset [40] is also available in three-channel RGB color of histopathology images. The biopsy tissues collected were stained with standard staining protocol, hematoxylin and eosin (H&E), which results in a total of 400 histopathology images.

### 2.3. Breast Cancer Image Classification using Deep Learning Approaches

In earlier studies, the classification of breast cancer images centralized on traditional machine learning methods such as Support Vector Machine (SVM) [41,42,43], Naïve Bayes [44,45,46], and Random Forest [47,48]. Machine learning involves the algorithms design and deployment to assess data and corresponding attributes without any prior task based on predetermined inputs from the environment [49]. Traditional machine learning methods rely on the quality of feature extraction that is limited to certain problems resulting from its shallow classifier [25]. Lately, the deep learning methods have been proven for more promising results specifically on large and complex data [50]. The implementation of feature learning methods (transfer learning) in deep learning helps to reduce the computational time, yet it obtained significant accuracy value compared to machine learning with hand-crafted features [51]. Generally, deep learning in CAD system outperformed the traditional approach because the automatic learning feature was created to analyze the variation and complexity of images directly; hence, convolutional neural network (CNN) is the most common model used for breast cancer diagnosis [52,53]. In 2020, Lin and Jeng [54] proposed a CNN model with uniform experimental design (UED) to classify breast cancer histopathology images. Their model outperformed other established deep learning models with lowest computational time. Current computing power can help to solve the related problems and further improve the quality of health and life among the community.

Deep learning is an established and emerging approach among researchers in the field of machine learning [55,56]. The main objective when employing deep learning is to discover multiple levels representations based on learning algorithm which are aimed for higher-level features for image classification and identification [50,57,58]. Generally, it is focused on learning algorithm that is able to learn, develop, and improve on its own to process data. Deep learning algorithms can extract features from high-dimensional images for internal representation [16]. Traditional machine learning works well with structured data with up to hundreds of features or characteristics. Unfortunately, for unstructured data, the analysis process will become tedious, or worse: unfeasible. Unstructured data are data stored in unstructured format and not prescribed by data models such as image, media, text data, and audio. Deep learning models different fundamental or needed qualities in data using a model architecture that is made up of different processing layers and non-linear variations [50,55,59].

It has been observed that researchers’ attention has recently shifted to deep learning because of its great success in solving problems related to unstructured data. Convolutional Neural Network (CNN) is a part of deep learning models that can be used for image classification and feature extraction effectively [60]. In the medical field, deep learning provides a useful approach for assisting radiologists in making an early breast cancer diagnosis with histopathology images [59,61]. Breast cancer classification, signal processing [62], and image analysis [63] have benefited from deep learning methods in recent years.

In 2021, Zuluaga-Gomez et al. [60] designed a deep learning architecture from CNN to detect patterns visually on thermal images (DMR-IR database). They proposed a Bayesian optimization, Tree Parzen Estimator (TPE), as the hyper-parameter to optimize the algorithm. Experimental results showed competitive improvement of the CNN approach with an accuracy of 92%. The study also proved that data pre-processing and data augmentation help in improving the model performance. Similarly, Alom et al. [63] presented a novel CNN approach based on inception and residual networks for breast cancer multi-classification with different data augmentation methods. The experiments showed improvement of accuracy by approximately 1.05% (image-level) and 0.55% (patient-level) as compared with models that were based on learning and were data-driven for multi-classification.

With the aim of detecting and identifying breast cancer, Hirra et al. [59] applied a patch-based deep learning approach, Deep Belief Network (DBN), for automatic features extraction on histopathology images. The proposed model, namely, Ps-DBN-BC, gained a promising result with an accuracy greater than 85%, hence outperforming the 17-layer CNN architecture. This work indicated that architecture with deeper layers does not necessarily provide outstanding performance. Hameed et al. [61] developed an ensemble deep learning approach for histopathology images to classify carcinoma or non-carcinoma images automatically. They used two pre-trained deep CNN-based models for excellent convergence results in a small dataset, and the accuracy obtained was 95.29%. On the other hand, deep learning also benefited the signal processing area, as presented by Pavithra et al. on the effectiveness of thermography for breast cancer detection with appropriate choice of feature extraction, segmentation, and classification algorithms [62].

## 3. Materials and Methods

### 3.1. Bibliometric Analysis

The implementation of bibliometric analysis at the beginning of the research process is popular among researchers because it helps to discover the information underlying the published articles in specific areas or topics [64,65]. Although there are different methods to explore and organize earlier findings from the literature search, bibliometrics has advantages in terms of being a systematic, understandable, and reproducible review process [66]. A detailed bibliometric analysis can capture the growth of particular research studies in a given time period [67]. The bibliometric networks were visualized using R Programming Language [68] and VOSviewer software [69]. This study executed co-authorship and co-occurrence analyses for network mapping. A bibliometric analysis was used to study the relationship of scientific publications among countries and authors by constructing and visualizing the network maps.

### 3.2. Data Collection

The data retrieval process involved the Scopus database, retrieved on 22 October 2021. Scopus is one of the largest relevant academic abstracts and indexing databases from Elsevier [30,70]. Scopus is also more effective for health-related topic searches compared to other databases such as PubMed and Web of Science [71,72]. The bibliometric analysis reviewed all related published articles between the year 2014 to 2021.

Articles included in the research focused on histopathology breast cancer images. For further analysis, articles that mentioned deep learning, convolutional neural network, transfer learning, breast cancer, breast neoplasm, breast tumor and breast diagnostic were included. The articles were selected based on the abstract reviewed. All articles are available for download, and non-English articles were excluded.

**Figure 1:**
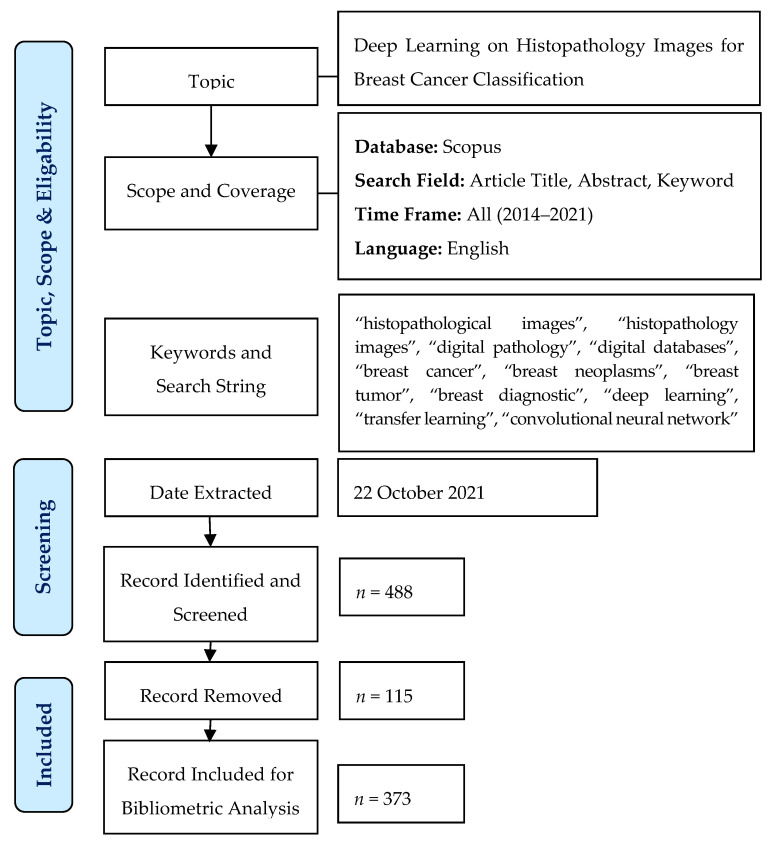
Flow diagram of the research process.

The study is focused on deep learning algorithms for breast cancer image classification. Hence, articles that use conventional neural networks or other machine learning techniques such as regression, clustering, and decision trees were excluded from this research. Deep learning algorithms have gained huge interest in biomedical image analysis [73,74]. In fact, deep learning algorithms have shown to be a better alternative for medical image classification and detection. There are several characteristics of deep learning such as incorporating a large amount of data, the depth of the network, and optimizing hyper-parameters. In addition, the study population involving other image types, for instance, mammogram, ultrasound, and thermogram, were discarded from the analysis. A total of 498 articles were extracted from the Scopus database. After the filtration process on inclusion and exclusion criteria, 488 articles were selected for elements extraction of the articles. After a thorough screening process based on the abstract, 373 articles were finally included for further analysis. Figure 1 shows the flow diagram of the research process.

## 4. Results and Discussion

### 4.1. Overview on Document and Source Type

Firstly, the data were tabulated based on the document type, where document type is referred to as a structured document with several valid elements and originality such as article, conference paper, review, and a book chapter. Meanwhile, source type is the source information for the documents, including journals, conference proceedings, and book series. There is a possibility of the abstracts from conference proceedings published twice as in the conference abstract and full journal [75]. Given the fast development in computer science and studies in the deep learning area, proceeding publications were also considered in this bibliometric analysis. Recent studies also showed that proceeding publications do have a significant impact on highly cited publications, especially in terms of citation counts [76,77]. The majority of the publications are scientific articles (48.53%), followed by conference papers (41.55%), conference reviews (4.02%), reviews (3.22%), and book chapters (1.87%) as shown in Table 2. Other document types represent less than 1% of the total publication.

### 4.2. Publication Growth

**Figure 2:**
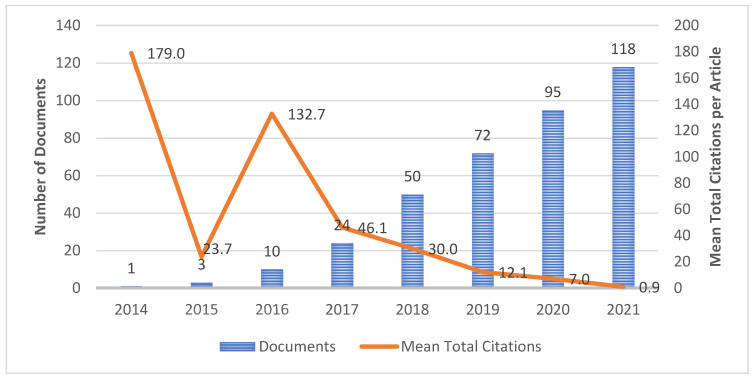
Flow diagram of the research process.

The pattern of publication growth is measured based on the published documents in the particular year. Figure 2 represents the publication trends and total mean citations of articles annually from 2014 to 2021. Scopus recorded Wang et al. [51] as the first published document on deep learning for breast cancer towards histopathology images in 2014, and to date, the document has more than 250 citations. Inspired by the rapid development of systems for invasive breast cancer detection, the authors combined a deep learning approach with hand-crafted features to maximize the model performance yet reduced the computational complexity since only light CNN method were implemented. They utilized 326 mitotic nuclei of breast cancer images in three-layer CNN architectures (two pooling layers and a fully connected layer). Since the number of images were low, they also used Synthetic Minority Oversampling Technique (SMOTE) to reduce the biasness during classification. Based on the comparison of several CNN-based methods, the results indicated that false positive (FP) errors were reduced, which showed that CNN was able to classify the images accurately. In 2016, one of the authors, Madabhushi A., collaborated with Janowczyk A. in [78] to analyze the digital pathology images using deep learning methods through segmentation and detection tasks of breast cancer images. They concluded that deep learning can be a reliable method because of the advantage in terms of feature extraction which can be directly extracted from the images. The study also has been cited by 747 documents since the first publication to date. This showed that more researchers are interested in deep learning-related research. Apart from that, Figure 2 also depicted the number of publications that increased steadily between 2015 and 2021, with the peak publications being in 2021, with 118 documents. This indicates the advancements in computing power and imaging technologies lead the researchers to explore the potential of deep learning to provide more promising results for histopathological image analysis [79,80,81]. From a citation perspective, the mean total citations of the documents were highest in 2014 and followed by year 2016; meanwhile, the lowest was for those published in 2021. This is not surprising as the citable years are not long enough after the publication [82].

### 4.3. Country Network Analysis

The co-authorship network of countries on breast cancer image classification using deep learning resulted in 71 countries from 2014–2021. Table 3 tabulates the top five countries according to their total link strength. The United States is considered a prominent country in scientific publications compared to others. The result is in line with other bibliometric analyses on “breast cancer” [33]. This could be contributed by greater financial support for researchers in the United States and the large population in the United States [83].

**Figure 3:**
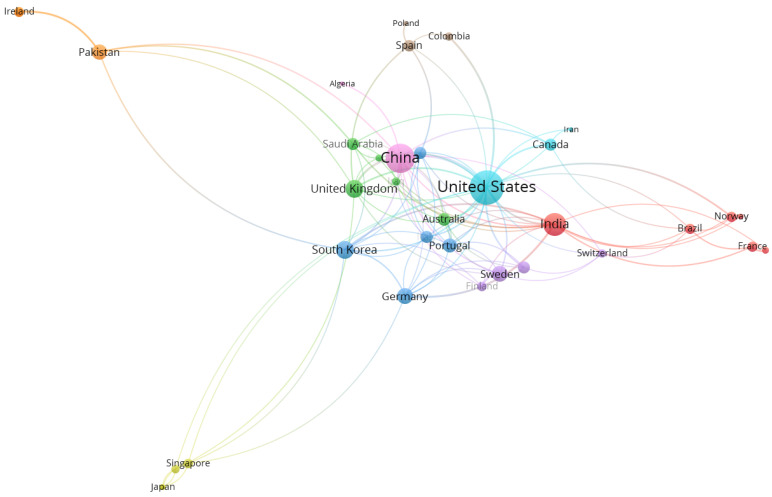
Co-authorship network visualization of countries in publication for 2014–2021.

Based on a threshold of three publications per country, 35 countries were matched as shown in Figure 3. The size of circle represents the total link strength and lines among the countries, representing the collaboration link between countries. In country network analysis, there are nine different colors which indicate a total of nine clusters formed (distinguished by the colors of red, green, blue, yellow, purple, aqua, orange, brown, and pink). For bibliometric analysis, normally each research constituent (countries, authors, and journals) was clustered using a combination of multidimensional scaling (MDS) and hierarchical clustering (see [84]). In this study, the clustering methods were based on a unified approach proposed by [85] with modularity-based clustering to explore the structure of the network such as social interaction among authors and their countries. For example, Cluster 6 (Aqua) has strong collaboration with other countries such as countries from Cluster 2 (Green) and Cluster 3 (Blue). All countries were connected to each other in the network map.

**Figure 4:**
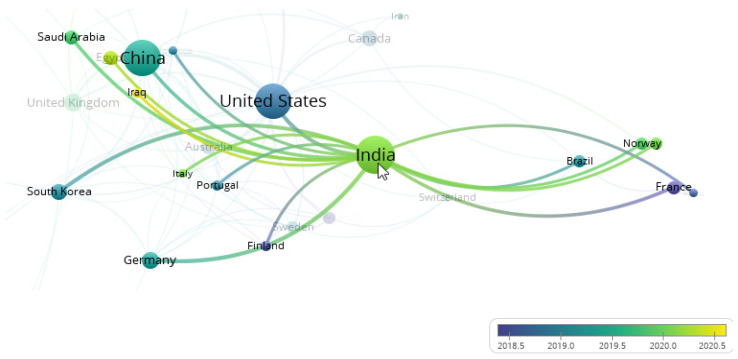
Co-authorship overlay visualization of India.

It is interesting to note that India is one of the countries with a high number of publications; however, the number of citations is far less than the United States and China. This could be explained by the passion of researchers to conduct studies on the topic within the country but the lack of collaboration with other countries. The overlay visualization in Figure 4 focuses on the country collaboration of India. There are total of 16 countries collaborated with India included Iraq, Norway, Saudi Arabia, South Korea, and France. By referring to the line that connected between each country to India, most of the countries started collaborated with India in early 2020. Deep learning methods have achieved great success in breast cancer image classification among researchers in India [86,87,88,89]. This also explains why the number of documents published in India is high but received lower citations, since the timeline between publication year and citable year is not long.

### 4.4. Author Network Analysis

**Figure 5:**
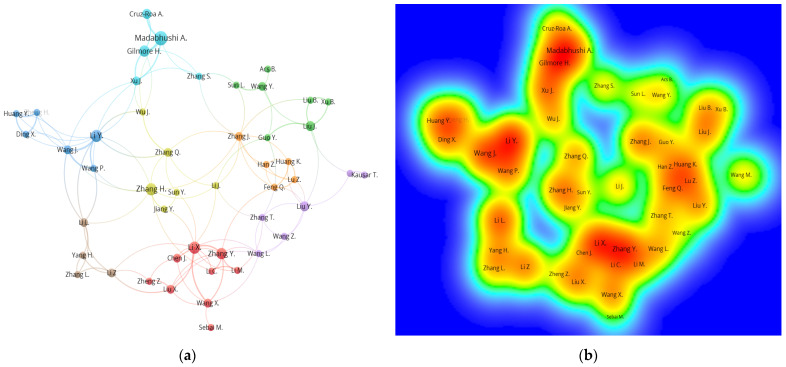
(**a**) Co-authorship network visualization of authors in publication for 2014–2021. (**b**) Density visualization of authors in publication for 2014–2021.

A total of 1310 authors published on the topic related to breast cancer image classification using deep learning. Among them, 9 authors (0.69%) published at least five documents, 39 authors (2.98%) contributed between three to four publications, and 1262 authors (96.34%) published at most two documents. From Figure 5a, the lines connected between authors shows their cooperation link. For example, a reasonable research link was indicated from close and strong interconnections between the collaboration of Zhang Y., Li X., and Wang X. from Cluster 1 (Red), Wang L. in Cluster 5 (Purple), and Li Z. in Cluster 8 (Brown). The authors Madabhushi, Gilmore, and Zhang S. in Cluster 6 (Aqua) were from the United States, while most authors from Cluster 1 (Red) represented authors from China. This indicated that authors from similar countries are closely linked and more likely to work together. Based on the density visualization (Figure 5b), Madabhushi A., Gilmore H., Li Y., Wang J., Li X., and Zhang Y. led the collaboration in breast cancer histopathology image research.

Table 4 presents the top 10 most productive authors ranked by the total link strength. It is interesting to note that the authors started to work collaboratively and contributed to publications after 2017; hence, the research domain has maintained its growth rate since. The total link strength of authors showed the collaboration closeness among them, which means higher total link strength indicated that more commonly collaboration occurs for the authors. An author from China, Li Y., had the most active collaboration with other authors such as Li L., Zhang H., Xu J., and Wang P., but the result showed that Madabhushi A. was the most highly cited author on the research topic. Some authors such as Xu J. and Gilmore H. had lower total link strength but recorded highly cited publications. This could be explained by referring to their popular publication related to nuclei detection using breast cancer histopathology images that has more than 600 paper citations [90].

Table 5 shows extra information on the research institutes and their research focus ranked based on the number of documents published in 2014–2021. The Case Western Reserve University has the highest number of publications that focuses on the convolutional neural network, digital pathology, image classification. Madabhushi A. from Case Western University has collaborated with authors from various institutes in all the nine documents published; hence, it is not surprising that Madabhushi A. has the highest paper citations. Out of 10 research institutes, four of them are in China while two in Canada and one each are in the United States, India, the Netherlands, and Sweden. This finding implies that convolutional neural network and deep learning-related research has improved in China over these years [91].

### 4.5. Journal Network Analysis

**Figure 6:**
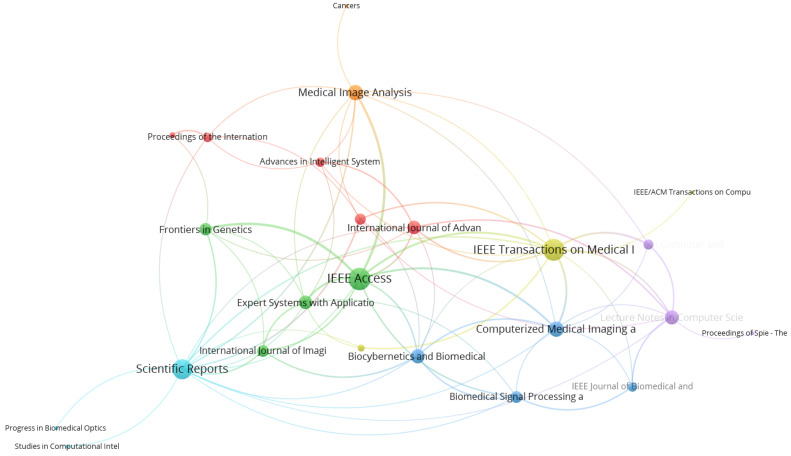
Citation network visualization of journals in publication for 2014–2021.

In journal network analysis, the number of articles published and the number of citations were considered while examining the most prominent journals in the topic of deep learning and breast cancer image classification. The citation analysis of journals resulted in 190 journals for 373 documents. Table 6 gives the top 20 journals published on breast cancer image classification using deep learning. Most publications in the related topic were published in Lecture Notes in Computer Science, Proceedings—International Symposium on Biomedical Imaging, IEEE Access, Scientific Reports and Communications in Computer and Information Science. Based on other indicators, IEEE Transactions on Medical Imaging and Scientific Reports have a significantly higher number of citations, with 703 and 451 citations, respectively. As shown in Figure 6, different node size represents different amounts of publications in the journal. Using a threshold of at least three articles per journal, only 24 journals were mapped in the network.

Research collaboration aims to combine various types of expertise for research output development by linking the knowledge and skills together. Co-authorship networks are commonly used to examine the collaboration patterns and discover the influential authors and organizations [92]. The analysis illustrates the social network structure that exists between individuals or organizations. Recently, the technological breakthrough in the CAD system has helped to improve the computational time of diagnosis and minimize the rate of misdiagnosis during image classification [93,94].

In the analysis, the involvement of the United States, China, and India as the most central countries in the network showed their scientific contribution to breast cancer and deep learning issues globally. The distance between each circle (node) implies the collaboration strength such that the further distance represents less collaboration between countries. Currently, the United States and China have contributed more than 40% of the total publications, and the collaboration strength between these countries is high. According to [34], a high number of publications in both countries are related to the investment of the business sectors in their Research and Development (R&D) expenditure. Apart from that, there is a growing trend of developing countries to engage in research related to the issues. For example, significant performance from China, India, and Pakistan is in line with previous studies that revealed breast cancer is among the important illness and research areas [95,96,97]. Both the developed and developing countries are publishing their research since breast cancer is a global burden issue [98].

Based on the density visualization, Madabhushi A. and Gilmore H. again are the most productive authors and most linked on the research topic. Many authors from various affiliations and countries collaborate with them such as Cruz-Roa A. from Universidad de los Llanos, Columbia [99] and Xu J. from Nanjing University of Information Science and Technology, China [100,101].

### 4.6. Co-Occurrence Analysis of Author Keywords

**Figure 7:**
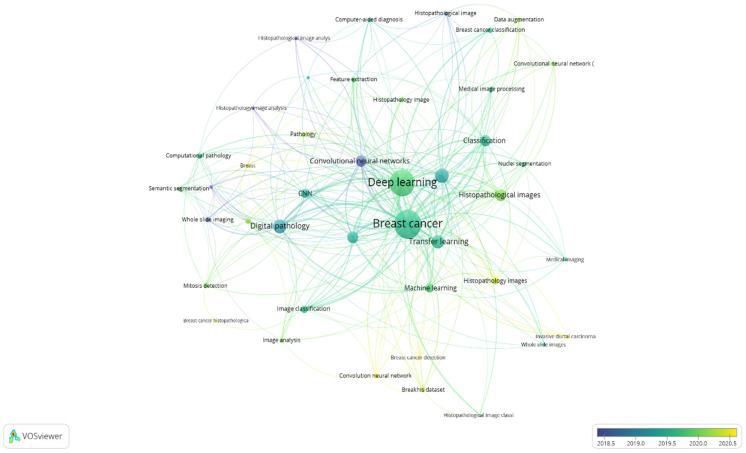
Co-occurrence overlay visualization of keywords in publication for 2014–2021.

For the years 2014 to 2021, a co-occurrence analysis of author keywords was conducted with a minimum of three keyword occurrences as a threshold for the study. Out of 657 keywords, 42 were found to be relevant. There are seven distinct clusters in the results (Figure 7). When two keywords appear together in one article or more, they are more likely to form a cluster. In Figure 7, the co-occurrence network map of keywords is depicted, given that the larger size of the circle, the higher the co-occurrence of keywords. Furthermore, having closer keywords together shows a stronger relationship. The average year of publication of the keywords was determined using colors. Notably, the focus of research from 2018 to 2019 was on biopsy image aspects (Dark blue) such as “histopathology image analysis”, “digital pathology”, “convolutional neural networks”, “whole slide images”, and “computer-aided diagnostics”. Instead, the network map reveals a greater focus on breast cancer classification approaches such as “deep learning”, “transfer learning”, “CNN”, “image classification”, “medical image processing”, and “feature extraction” from 2019 to date.

The top keywords that are identified through co-occurrence analysis is breast cancer and deep learning with 152 and 139 total number of counts, respectively. The result is as expected since breast cancer and deep learning are part of the search keywords for bibliometric analysis. Breast cancer studies received high attention in research related to deep learning. According to Samb et al. [102], chronic illness will lead to 80% human deaths by 2023, which also contributes to global issues, and proper treatments that are aimed at combating the illness may benefit the healthcare system. Specifically, breast cancer is also one of the current leading cause of deaths where the mortality rate is still high, even though the mortality trend has been reduced since 1989 [3]. Hence, researchers focus their work on early detection of breast cancer through deep learning technology [103,104]. This is supported by the overlay visualization in Figure 7, where the research direction aimed at the efficiency of CNN towards image analysis from 2018 to 2020. In 2018, Cruz-Roa et al. [99] proposed a new method based on CNN for histopathology image analysis on whole slide images. They applied the adaptive sampling technique to overcome issues on larger sizes of images.

Spanhol [12] said that a well-described image database is important for CAD system research, and a new histopathology image dataset known as BreakHis is introduced together with some experimental results using CNN models. Meanwhile, in 2019, Ghosh et al. [88] studied on deep learning and image segmentation and revealed that the medical imaging field needs various segmentations such as nuclei segmentation for reliable CNN performance. Alom et al. [63] proposed an Inception Recurrent Residual Convolutional Neural Network (IRRCNN) model based on several criteria such as magnification factors, image resizing, and image augmentation and segmentation. Result showed that the IRCNN model outperformed the state-of-the art method in 2016 using BreakHis dataset. In 2020, Salama et al. [105] introduced a hybrid deep learning method for breast cancer detection using pre-trained models, ResNet50 and VGG16. Theoretically, a promising accuracy rate depends on the amount of data for model training such that a large volume of training samples leads to a better accuracy rate. Since medical images have a limitation on the sample size, she addressed the limitation by utilizing a data augmentation technique and transfer learning which revealed that hand-crafted features and human interface can be discarded. A hybrid ResNet15 model achieved the highest accuracy, 97.98%, as compared to hybrid VGG16 and other models. However, for this deep learning algorithm to be fully established and exploited on a worldwide scale, significant challenges must be overcome. Some discussion on the challenges of deep learning for breast cancer classification using histopathology images is provided in the next section.

### 4.7. Computational Method for Histopathology Images

In recent years, there has been a growth and development in the use of deep learning algorithms for histopathology image analysis, specifically CNN methods. CNN methods could be used for identifying regions of interest (ROIs), feature extraction, and image classification. The advent of digital histopathology images with CNN methods offers tremendous potential for assisting pathologists with their jobs. Thereby, Table 7 shows a summary of some deep learning algorithms based on CNN methods in histopathology images. The five listed references are from a high-impact journal with over 100 citations.

Cruz-Roa et al. [106] aimed to assess the accuracy and reliability of deep learning algorithms for classifying the digital images into invasive tumor. They offered a novel method for classifying the invasive tumor on whole-slide images using a CNN-based method. In this study, classification performance was assessed across all the images retrieved from the Cancer Institute of New Jersey (CINJ) in the form of whole-slide images. They used three different convolutional network (ConvNet) layers—three-layer ConvNet, four-layer ConvNet, and six-layer ConvNet—as the classifier and compared them with handcrafted features (color, shape, texture, and topography). They concluded that the classification performance related with those features are lower and resulted in higher inconsistency as compared to the ConvNet classifier. Meanwhile, in mitosis detection analysis, the use of handcrafted features solely may result in low accuracy model, whereas CNN methods have issue on high computational cost. Hence, motivated from these drawbacks of handcrafted features and CNN methods, Wang H. et al. [51] introduced a hybrid approach for mitosis detection on ICPR12 dataset. To address these issues, handcrafted features and a CNN method are combined through cascaded ensemble. The results demonstrate that the accuracy of the provided approach still needs to be improved, and a GPU should be used to create a deep multilayer CNN model. Han Z. et al. [24] presented a breast cancer multi-classification technique that makes use of a deep learning model. They implemented a complete recognition approach based on a newly developed class-structure-based deep convolutional neural network (CSDCNN) to provide a consistent and accurate solution for breast cancer classification. They also utilized multi-scale data augmentation and over-sampling approaches to overcome overfitting and unbalanced classes issues. On a large dataset, the proposed CNN model performed admirably.

In Ghosh S. et al. [88], they stated CNN as among the most widely used methods in computer vision. For the segmentation tasks, CNN methods have undergone many basic adjustments to perform effectively. In addition, back-propagation enabled CNN to train a cascaded set of convolutional kernels. It has been greatly improved since then. Generally, they stated that the speed and accuracy of models are important factors in performance evaluation. The speed may be increased through network compression by using depth-wise separable convolutions, kernel factorizations, and a smaller number of spatial convolutions approaches. The popularity of generative adversarial networks (GANs) has recently risen, but there is still some room for improvement in image segmentation. A study by Alom M. Z. et al. [63] demonstrated how deep learning has outperformed state-of-the-art approaches in medical imaging areas. They developed an approach for breast cancer classification known as Inception Recurrent Residual Convolutional Neural Network (IRRCNN) model. This sophisticated DCNN model combines the strengths of the Inception-v4, ResNet, and recurrent CNN (RCNN) with several criteria on data augmentation techniques. Compared with other relevant deep learning algorithms such as inception, RCNN, and residual network, the IRRCNN model offers better performance while utilizing the same or less network parameters.

### 4.8. Challenges and Future Directions

In this bibliometric analysis, we discovered that deep learning algorithms can be utilized to classify breast cancer histopathology images, given that the model performance (in terms of accuracy) is equal or better as compared to healthcare professionals. However, some parameters must still be considered for a reliable and consistent output. With so much focus on the advancement of deep learning, more individuals are interested in its performance in healthcare.

#### 4.8.1. Large Image Size

In deep learning, image classification frequently utilized small-sized images as an input for the network. Large images have to be resized to fit the network requirement given that a larger size of images leads to a large amount of parameter estimation, computational power, and memory usage. In analysis, whole slide images (WSI) are commonly difficult to be examined, but resizing the images could reduce the information of the cell which leads to less accurate image classification. Therefore, the WSI is often divided into patches (small regions) so that each patch can be evaluated independently. Recently, the findings from Zhou L. et al. [107] demonstrate the benefits of using CNN methods to classify the breast images patch by patch, and the assessment of breast imaging information may yield more accurate and reproducible imaging diagnoses than human interpretation.

#### 4.8.2. Color Variations

For comparable results during analysis, color variation is another issue in deep learning models. Different batches or manufacturers of staining solutions, thickness of tissue sections, staining settings, and scanner models are all sources of variance [49]. Learning without taking color variation into account may degrade the performance of deep learning models. Several techniques have been proposed to deal with the color variation of the images such as color augmentation, color normalization, and grayscale conversion [49]. Grayscale conversion is the simplest method [59], but it may be overlooking critical information on the color representation commonly used by pathologists. Color normalization attempts to change the color values of an image pixel by pixel, using some methods such as color constancy, color deconvolution, and color transfer. Color normalization could be appropriate when the images have identical cell or tissue compositions. However, the utilization of color normalization should be handled carefully because it may reduce the accuracy of the classification algorithm related to histopathology images [108].

#### 4.8.3. Insufficient Data

When there is insufficient data, usually CNN models are less generalized and may lead to an overfitting problem. One approach to avoid the issue is through data augmentation tasks which helps to increase the performance of CNN models in image classification. Recently, automatic approaches to data augmentation, such as data augmentation based on multi degree-of-freedom (DOF) automatic image acquisition, have been presented by Chen L. et al. [109]. It is necessary to assess the physical validity of the created samples and the implications of the several generated problems on the algorithm performance. Several methods for generating synthetic samples using generative adversarial networks have recently been proposed Zhou F. et al. [110]. The generative adversarial network can generate samples in data augmentation tasks rapidly, especially in image-to-image translation [20].

## 5. Conclusions

The bibliometric analysis study highlights the growing trend of breast cancer and deep learning research globally. This study conducted a bibliometric analysis and visualization of breast cancer image classification using deep learning publications from 2014 to 2021. This study examined some noteworthy findings connected to the related publications. The topic of breast cancer image classification using deep learning has seen a lot of research over the last eight years, with the publications output growing at an exponential rate since 2014. There is a growing interest in breast cancer and deep learning research, which is in response to the pressing demand for urban growth and quality of life. With the technological advancements that have occurred in the last two decades, tremendous progress has been noticed in breast cancer and deep learning studies across all disciplines [93,105].

The main study areas in the realm of breast cancer image classification using deep learning could be recognized based on co-keyword network analysis: (i) breast cancer; (ii) deep learning; (iii) convolutional neural network; (iv) digital pathology; and (v) transfer learning. The theme of the study changed swiftly as time went on, and several fields of breast cancer image classification using deep learning research were thriving at the same time, according to keyword bursts analysis. The histopathology images, invasive ductal carcinoma, and BreakHis dataset have all become new research centers. About 98.54% of authors (*n* = 1291/1310) were credited in not more than three papers on the issue of breast cancer image classification using deep learning, according to co-authorship analyses. This could indicate that a substantial percentage of authors were new to the field of research. Author collaboration network analysis revealed that Li Y., Madabhushi A., and Gilmore H. were among the most productive authors, the most linked authors, and the most cited authors. This suggests that those authors are pioneers in the field of research. Over the past eight years, deep-learning-related methods, especially CNN, have showed outstanding performance in breast cancer image classification. However, data related to medical images or microscopy images are normally limited due to a small number of patients. A large amount of data is required for training the model effectively. Therefore, some researchers used image segmentation techniques to overcome the problem. Data augmentation can help to increase the number of input images by adding copied images from the original input. The new images are slightly modified using several data augmentation strategies such as rotation, flipping, and scaling.

In this study, some challenges related to the CNN method are discussed, and data insufficiency might be the biggest challenge in medical data for image classification. This is also supported by Komura and Ishikawa [49]: their work stated that a large amount of training data is important for image classification tasks. A vast amount of research has been conducted on CNN methods with several adjustment to reach model efficiency of image classification specifically on breast cancer histopathology images. As discussed in the previous section, recently, some studies revealed that generative adversarial networks (GANs) could be used to generate samples for training datasets, so that issue on data scarcity can be tackled. The implementation of GANs in future studies as a data synthesis option should be further explored to elevate the computational time and improve the performance of the CNN methods.

VOSviewer used country collaboration analysis to divide the 35 countries into nine research strong-linked clusters, led by the United States, China, India, South Korea, and the United Kingdom, respectively. They were also at the forefront of a collaborative effort to classify breast cancer images using deep learning. The United States and China were both ranked in the top two in author collaboration and country collaboration analyses. China, on the other hand, has recently adopted a more cooperative attitude. In fact, China is one of the world’s newest scientific hubs.

This bibliometric study has some limitations to be addressed. First, the data collection was restricted to Scopus’ core collection, with improvements such as “source type” and “languages” being used. Other databases such as PubMed or WoS should have been combined as well. Nonetheless, Scopus is one of the world’s largest and most utilized databases for scientific publication analysis, particularly in the healthcare area. Second, since some recently published papers have low citation frequency, there may still be discrepancies between true research status and our bibliometric analysis results [111]. As a conclusion, the role of deep learning in breast cancer image classification will keep evolving. However, deep learning is not a replacement for pathologists; instead, it will continue to assist them with tools that are both effective and efficient. This bibliometric analysis could be used as a springboard for more specific and in-depth research.

## Figures and Tables

**Table 1:** Summary of image distribution for different magnification factors.

Magnification	40×	100×	200×	400×
Benign	652	644	623	588
Malignant	1370	1437	1390	1232

**Table 2:** Document type from Journal.

Document Type	Frequency	Percentage (*n* = 373)
Article	181	48.53
Conference paper	155	41.55
Conference review	15	4.02
Book chapter	7	1.87
Erratum	1	0.27
Note	1	0.27
Review	12	3.22
Short Survey	1	0.27
Total	373	100.00

**Table 3:** Document type from journal.

Country	TLS ^1^	Links	Documents	Citations	Cluster
United States	51	21	68	2235	6
China	39	17	71	1586	9
India	26	16	80	674	1
South Korea	17	11	12	444	3
United Kingdom	17	10	20	190	2
Germany	14	10	14	239	3
Sweden	14	9	10	192	5
Pakistan	13	6	13	172	7
Portugal	12	10	5	157	3
Australia	10	7	13	201	2

^1^ Total link strength.

**Table 4:** Document type from journal.

Author	TLS ^1^	Links	Documents	Citations	Affiliation	APY ^2^
Li Y.	18	10	7	93	Chongqing University, China	2018
Madabhushi A.	15	5	9	912	Case Western Reserve University, United States	2017
Li X.	15	10	7	56	Chongqing University of Posts and Telecommunications, China	2020
Wang J.	14	8	4	31	Chongqing University, China	2020
Gilmore H.	13	5	6	891	Case Western Reserve University, United States	2017
Zhang Y.	13	8	6	38	Nanjing University, China	2019
Li. L	13	7	4	36	Chongqing University, China	2020
Xu J.	11	7	4	485	Nanjing University, China	2018
Zhang H.	10	9	7	130	East China Jiaotong University, China	2019
Li Z.	10	6	4	22	Chongqing University of Posts and Telecommunications, China	2020

^1^ Total link strength; ^2^ average publication year.

**Table 5:** Research institutes and their research focus.

Affiliation	Research Focus	Document
Case Western Reserve University	Convolutional neural network, digital pathology, image classification	9
Indian Institute of Technology Kharagpur	Features, convolutional neural network, whole slide images	7
Shenzhen University	Image classification, convolutional neural network	6
Radboud University Medical Center	Deep learning, whole slide images	6
University of Toronto	Convolutional neural network, review analysis	6
Karolinska Institute	Convolutional neural network, classification, deep learning	5
Xiamen University	Segmentation, detection, convolutional neural network	5
Sunnybrook Health University	Deep learning-based, convolutional neural network, feature extraction	5
Southern Medical University	Deep learning, cancer staging, classification	4
Chongqing University	Features, convolutional neural network, image classification	3

**Table 6:** Top 5 journals in publication for 2014–2021.

Journal	TLS ^1^	Links	Documents	Cit ^2^
IEEE Access	26	10	10	48
IEEE Transactions on Medical Imaging	24	13	5	703
Scientific Reports	21	16	10	451
Computerized Medical Imaging and Graphics	14	9	4	114
Medical Image Analysis	14	10	5	177
Biocybernetics and Biomedical Engineering	12	8	5	41
Lecture Notes in Computer Science (including subseries lecture notes in artificial intelligence and lecture notes in bioinformatics)	12	8	36	433
Expert Systems with Applications	11	8	3	117
International Journal of Advanced Computer Science and Applications	11	7	4	66
Frontiers in Genetics	10	6	3	71
Biomedical Signal Processing and Control	9	6	5	25
International Journal of Imaging Systems and Technology	9	5	6	27
Journal of Medical Imaging	8	6	3	249
Communications in Computer and Information Science	7	4	10	10
Advances in Intelligent Systems and Computing	6	5	8	16
IEEE Journal of Biomedical and Health Informatics	6	4	6	101
Proceedings of the International Joint Conference on Neural Networks	6	5	4	417
Proceedings—International Symposium on Biomedical Imaging	4	3	13	262
Lecture Notes in Electrical Engineering	3	2	3	0
Cancers	1	1	6	9

^1^ Total link strength; ^2^ citations.

**Table 7:** Five references in publication in high impact journal on CNN methods.

References	Journal	Model/Method	IF ^1^	H-Index	Cit ^2^	Year
Cruz-Roa et al. [106]	Scientific Reports	CNN/ConvNet	4.380	213	292	2017
Wang H. et al. [51]	Journal of Medical Imaging	CNN and handcrafted features	3.610	29	272	2014
Han Z. et al. [24]	Scientific Reports	Structured based deep CNN	4.380	213	210	2017
Ghosh S. et al. [88]	ACM Computing Surveys	Deep learning, CNN	10.282	163	126	2019
Alom M. Z. et al. [63]	Journal of Digital Imaging	Deep CNN, Inception-v4, ResNet, Recurrent CNN	4.056	58	123	2019

^1^ Impact factor; ^2^ citations.

## Data Availability

The data presented in this study are available on request from the corresponding author.
